# Association between Variants of the Autophagy Related Gene – IRGM and Susceptibility to Crohn’s Disease and Ulcerative Colitis: A Meta-Analysis

**DOI:** 10.1371/journal.pone.0080602

**Published:** 2013-11-13

**Authors:** Xiao Cheng Lu, Yi Tao, Chen Wu, Peng Lai Zhao, Kai Li, Jin Yu Zheng, Li Xin Li

**Affiliations:** Department of Neurosurgery, First Affiliated Hospital of Nanjing Medical University, Nanjing, Jiangsu, China; Massachusetts General Hospital, United States of America

## Abstract

**Background:**

Polymorphisms in immunity-related GTPase family M (*IRGM*) gene may be associated with inflammatory bowel disease (IBD) by affecting autophagy. However, the genetic association studies on three common variants in *IRGM* gene (rs13361189, rs4958847 and rs10065172) have shown inconsistent results.

**Methodology/ Principal Findings:**

The PubMed and Embase were searched up to June 5, 2013 for studies on the association between three *IRGM* polymorphisms and IBD risk. Data were extracted and the odd ratios (ORs) and 95% confidence intervals (95% CIs) were calculated. Finally, we performed a meta-analysis of 25 eligible studies in 3 SNPs located at *IRGM* gene by using a total of 20590 IBD cases and 27670 controls. The analysis showed modest significant association for the rs13361189, rs4958847 and rs10065172 variants in Crohn’s disease (CD): the risk estimates for the allele contrast were OR=1.306 (1.200-1.420), p=5.2×10^-10^, OR=1.182 (1.082-1.290), p=0.0002, and OR=1.248 (1.057-1.473), p=0.009 respectively (still significant when the p value was Bonferroni adjusted to 0.017). When stratified by ethnicity, significantly increased CD risk was observed in Europeans, but not in Asians. Conversely, there was no association of rs13361189 or rs4958847 variant with risk of ulcerative colitis (UC).

**Conclusions/ Significance:**

These results indicated that autophagy gene-*IRGM* polymorphisms appear to confer susceptibility to CD but not UC, especially in Europeans. Our data may provide further understanding of the role of autophagy in the pathogenesis of CD.

## Introduction

Inflammatory bowel disease (IBD), a chronic inflammatory disease of the gastrointestinal tract, is usually classified into two clinical forms: Crohn’s disease (CD) and ulcerative colitis (UC) [1,2]. CD generally involves the ileum and colon, and it can affect any region of the intestine in a continuous manner. UC involves the rectum and may affect part of the colon or the entire colon, often uninterruptedly. The etiology of IBD most likely involves a complex interaction of genetic and environmental factors. Although the etiology remains poorly understood, epidemiologic and linkage studies suggest that genetic factors are implicated in the pathogenesis of IBD [3-9]. 

Recent progress in the genetics of IBD has advanced understanding of disease pathogenesis. GWAS meta-analysis identified 71, 47 and 163 susceptibility loci of CD, UC and IBD, respectively. These genes involved in intestinal barrier function (GNA12 and LAMB1), transcriptional regulation (NKX2-3 and IRF5) and immune response (IL23R and IL12B). Recently, studies in animal models and IBD patients suggested that autophagy related genes (*ATG16L1* and *IRGM*) may play an important role in the pathogenesis of IBD[10–12]. The immunity-related GTPase family M (*IRGM*) gene, located on chromosome 5q33.1, encodes a GTP-binding protein that induces autophagy, which is involved in elimination of intracellular pathogens[13,14]. Association of three common polymorphisms in *IRGM* gene (rs13361189, rs4958847 and rs10065172) with IBD has been recently reported [12,15-18]. However, the genetic association studies that investigated the association between IBD and rs13361189, rs4958847 or rs10065172 variant have produced inconclusive results. For instance, an accumulating number of studies suggested a positive association between *IRGM* polymorphisms and CD susceptibility [12,18,19], which, nevertheless, could not be replicated in several studies [16,20,21]. This inconsistency may be due to studies with limited sample sizes, inadequate statistical power, or ethnic differences. 

Meta-analysis is a proper method to deal with these ambiguities and overcome the problem of small sample sizes and inadequate statistical power in different genetic studies. In the present study, we performed a meta-analysis of all eligible case control and cohort studies to clarify the associations between three common polymorphisms (rs13361189, rs4958847 and rs10065172) in the *IRGM* gene and IBD (CD or UC) susceptibility. 

## Materials and Methods

### Identification and eligibility of relevant studies

Electronic searches in Medline, Embase, CNKI (China National Knowledge Infrastructure) and Chinese Biomedicine databases were performed using the following search terms: ‘Inflammatory Bowel Disease’ or ‘IBD’, ‘Crohn’s disease’ or ‘CD’, ‘ulcerative colitis’ or ‘UC’, ‘*IRGM*’, ‘polymorphism’ or ‘variant’, ‘rs13361189’, ‘rs4958847’ or ‘rs10065172’, and ‘single nucleotide polymorphism’ or ‘SNP’( the last search update was 5 June 2013). In addition, the reference lists of all retrieved articles were checked for additional potential studies. A study was included in the analysis if (1) reported the relationship between the polymorphisms of IRGM rs13361189, rs4958847 or rs10065172and the risk of IBD; (2) the genetic information of included studies was from unrelated populations (studies of which the design was not based on family data). Major reasons for exclusion of studies: (1) no control population; (2) studies that contained overlapping data; (3) comments, letters, review articles, or articles only with an abstract. Additionally, when a study reported the results on different subpopulations or panels, we treated them as separate studies in the meta-analysis.

### Data extraction

The following data was extracted independently from each study by two authors: first author, journal, year of publication, country of origin, ethnicity of the individuals involved (Europeans, Asians, or Africans), genotype frequency, sex and mean age in cases and controls. Of the studies with the overlapping data of the same population resource, we selected the most recent ones with the largest number of participants. If the article did not provide sufficient genotype distribution, the corresponding author was contacted for the detailed data. In addition, disagreements were resolved by discussion between the two investigators.

### Statistical analysis

The strength of the association between *IRGM* polymorphisms and CD or UC risk was evaluated by the odds ratios (ORs) with 95% confidence intervals (CIs). For the rs13361189 polymorphism, we examined the allelic effect of C (minor allele) versus T (common allele), and also examined the contrast of CC versus TT, CT versus TT, CC+CT versus TT (dominant model), as well as CC versus CT+TT (recessive model). Similar models were analyzed for the rs4958847 and rs10065172 polymorphisms. The association between rs10065172 and UC risk was not evaluated for the lack of sufficient data. The significance of the pooled OR was determined by the Z-test; and the P values were adjusted using Bonferroni correction by the number of compared SNPs. (P=0.05/3=0.017) In addition, for each genetic contrast, stratified analysis was performed according to ethnicity. The Hardy-Weinberg equilibrium (HWE) in the control group was assessed, and P<0.05 was considered as significant disequilibrium.

The heterogeneity between studies was assessed by Chi-square based Q test [22] and I^2^ test. Heterogeneity was considered significant when P<0.10, and then the random effect model was applied for meta-analysis, otherwise, a fixed-effects model was used [23]. I^2^ takes values between 0% and 100% with higher values denoting a greater degree of heterogeneity [24]. In addition, a meta-regression model was performed to explore the possible heterogeneity among different kinds of studies. 

Cumulative meta-analyses were carried out for all three variants in association with CD and two variants (rs13361189 and rs4958847) with UC to evaluate the trend of the genetic risk effect (OR) of the allele contrast as evidence accumulating over time. To assess the stability of the results, sensitivity analysis was carried out after sequential removal of each study or by excluding those studies deviated from HWE.

Publication bias was investigated using graphical evaluation of funnel plots. However, the funnel plot may be not considered strictly as a test of publication bias. Then, the Egger’s test was used to provide statistical evidence of funnel plot symmetry [25]. If significant publication bias was detected, ORs and 95% CIs would be adjusted by trim and fill methods. All statistical analyses were performed by STATA software (version 12).

## Results

### Main characteristics of eligible studies

**Figure 1:**
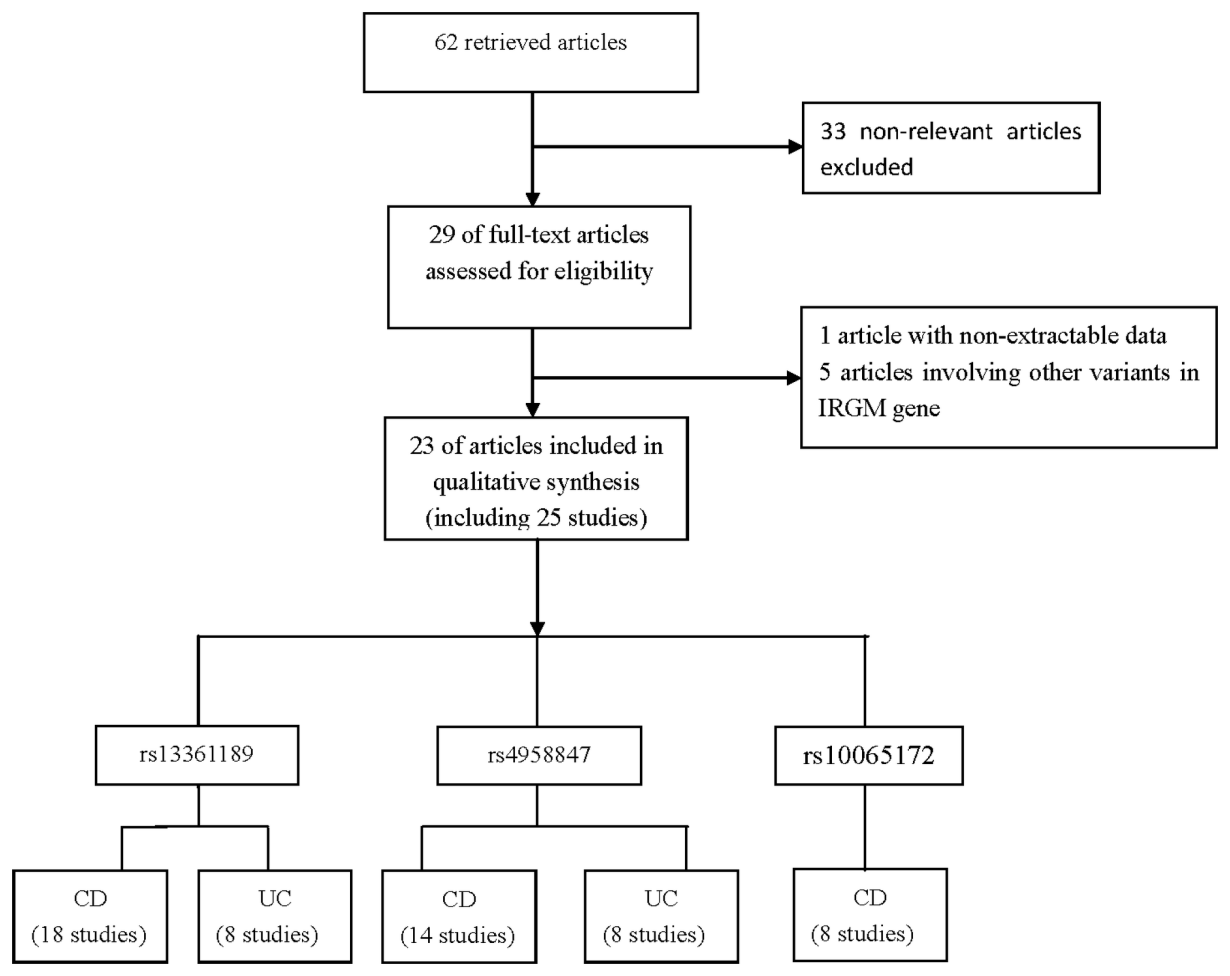
Flowchart of search strategy for meta-analysis.

The literature review identified 62 articles in PubMed, Embase, CNKI and Chinese Biomedicine databases that met the search criteria. The abstracts and full articles of the retrieved studies were read to assess their appropriateness for meta-analysis. Finally, a total of 23 relevant articles with *IRGM* polymorphisms (rs13361189, rs4958857 or rs10065172) and IBD (UC or CD) were included in this meta-analysis. Figure 1 showed a flow chart of the retrieved studies and the excluded studies. Among them, one publication [16] contained data on two different subpopulations, one [18] included Wellcome Trust Case Control Consortium (WTCCC) samples and replication Crohn's disease (RCD) samples and we treated them independently. Therefore, 25 studies that comprised a total of 20590 IBD cases and 27670 controls were considered in our meta-analysis. 

A list of details of the studies included in the meta-analysis was provided in Table 1. The studies were published from 2007 to 2013. Studies were conducted in various populations of ethnic descent: 17 Europeans [12,15-19,21,26-35], 4 Asians [36–39], 1 Africans [20] and 1 Jewishs [40]. Because of the insufficient samples available for African and Jewish groups, we have performed subgroup analysis in Europeans and Asians. Although the allele frequency of the *IRGM* polymorphisms was extracted from 25 studies, 4 studies [19,28,30,31] did not include genotype distributions, but one [19] was included after we contacted the authors directly, who provided sufficient data. In 2 studies [18,21], the distribution of the genotypes in control group was not in HWE (P<0.05). Then, a sensitivity analysis was performed by excluding these studies from the analysis.

**Table 1:** Characteristics of Studies Included in the Meta-analysis.

				Cases				Controls			
Author, Year of publication	**Ethnicity**	**IRGM variant **	**Phenotype Studied **	**Number**	**Males (%) **	**Age or Age at diagnosis**		**Number **	**Males (%) **	**Age**	**Matching**
Wang, 2012	African	rs13361189	CD	CD:354	34.2	37.4±14.3 and 26.7±12.9 at diagnosis		354	42.6	39.8±12.7	nr
		rs4958847									
		rs10065172									
Meggyesi1, 2010	European	rs13361189	CD and UC separately	CD: 456 UC: 274	CD:53.6	CD: 37.1±12.6 and 26.5±10.6 at diagnosis		271	nr	nr	Age and sex
Meggyesi2, 2010	European	rs13361189	CD and UC separately	CD:352 UC: 154	UC:47.2	UC: 43.7±15.0 and 31.3±13.4 at diagnosis		198	nr	nr	
Peter, 2011	Jewish	rs13361189	CD	CD:369	nr	nr		503	nr	nr	nr
Dema, 2009	European	rs4958847	CD	CD:725	nr	nr		956	nr	nr	nr
		rs10065172									
Frank, 2008	European	rs13361189	CD and UC separately	CD: 1850	CD:32	CD: 38 and 21 at diagnosis (median)		1817	nr	nr	Age and sex
		rs4958847		UC: 1103	UC:42.8	UC: 40 and 26 at diagnosis (median)					
		rs10065172									
Wolfkamp, 2010	European	rs13361189	CD	CD: 530	nr	nr		529	nr	nr	nr
		rs4958847									
Palomino-Morales,2009	European	rs13361189	CD and UC separately	CD: 557	nr	nr		672	nr	nr	Age and sex
		rs4958847		UC: 425							
Yamazaki, 2009	Asian	rs13361189	CD	CD: 484	72.5	22.4(7–55)		470	50.2	38.7(21–77)	nr
		rs4958847									
Prager, 2012	European	rs13361189	CD and UC separately	CD: 464	CD:37.5	CD:29.5±11.6 at diagnosis		508	42	60±16.2	nr
		rs4958847		UC: 292	UC:44.8	UC:34.3±14.2 at diagnosis					
Fisher, 2008	European	rs13361189	UC	UC: 1841	nr	nr		1470	nr	nr	nr
		rs4958847									
Parkes (RCD), 2007	European	rs13361189	CD	CD:1182	40.3	CD: 43.9 and 25.5 at diagnosis (median)		2024	nr	nr	nr
		rs4958847									
Parkes (WTCCC), 2007	European	rs13361189	CD	CD:1748	39.2	CD: 45.7 and 26.1 at diagnosis (median)		8655	nr	nr	nr
		rs4958847									
Roberts, 2009	European	rs13361189	CD and UC separately	CD: 507	nr	nr		576	nr	nr	nr
		rs4958847		UC: 475							
Latiano, 2009	European	rs4958847	CD and UC separately	CD: 823		CD: 30 ± 15 at diagnosis		578	nr	nr	nr
				UC: 353		UC: 25 ± 16 at diagnosis					
Zheng, 2012	Asian	rs13361189	CD	CD:318	48.4	CD: 37.2±11.4		318	49.1	36.7±12.3	nr
Prescott, 2010	European	rs13361189	CD	CD:1848	nr	nr		2025	nr	nr	nr
		rs10065172									
Pang, 2011	Asian	rs13361189	CD	CD:66	48.5	CD:36.3±11.8		66	50.0	35.4±13.1	nr
Limbergen, 2009	European	rs13361189	CD	CD:630	nr	nr		3283	nr	nr	nr
		rs4958847									
		rs10065172									
Weersma, 2009	European	rs13361189	CD and UC separately	CD: 1656	nr	nr		1086	nr	nr	nr
		rs4958847		UC: 1075							
Eglinton, 2012	European	rs4958847	CD	CD: 507	37.1	45±17.9		507	nr	nr	nr
Amre, 2009	European	rs10065172	CD	CD: 289	55.4	CD: 12.1± 3.5 at diagnosis		290	52.4	11.4± 6.8	nr
Duraes, 2013	European	rs13361189	CD	CD: 511	46.2	CD: 28.6± 11.2 at diagnosis		626	38.5	30.5(9-83)	sex
Glas, 2013	European	rs13361189	CD and UC separately	CD: 817	CD: 46.0	CD: 40.7±13.3 and 27.9±12.0 at diagnosis		961	63.6	47.4±9.06	nr
		rs4958847		UC: 283	UC: 53.0	UC: 43.8±14.4 and 31.3±13.7 at diagnosis					
		rs10065172									
Moon, 2013	Asian	rs4958847	CD and UC separately	CD: 253	CD:60.9	CD:25.9±10.4 at diagnosis		520	56.5	39.3±15.8	Age and sex
		rs10065172		UC: 257	UC:50.6	UC:37.1±12.4 at diagnosis					

CD: Crohn’s disease, UC: ulcerative colitis, nr: not report.

### Quantitative synthesis

#### Crohn’s disease

**Figure 2:**
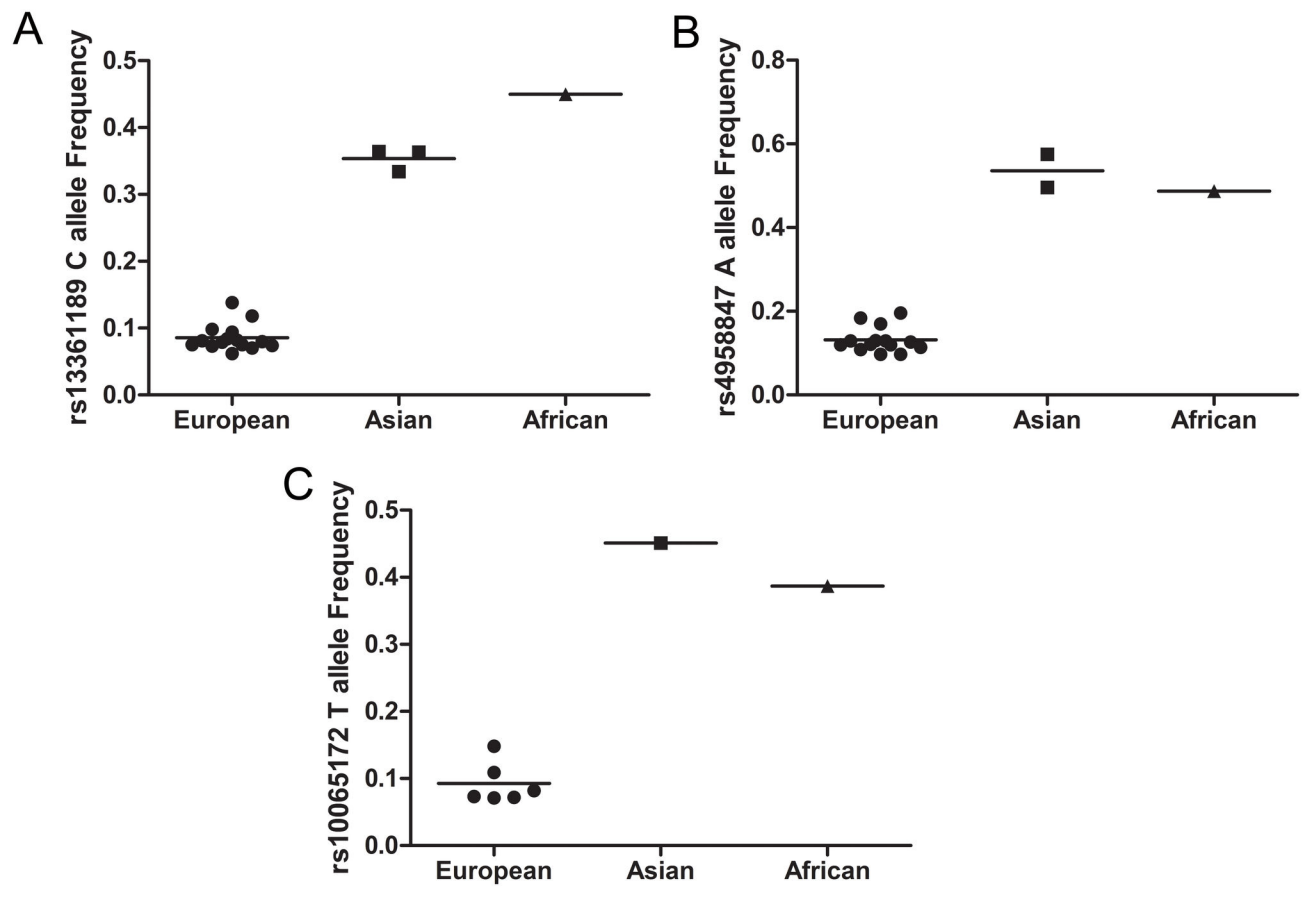
Allele frequencies (%) in the three major ethnical groups in controls of CD to (A) rs13361189, (B) rs4958847 and (C) rs10065172. Each data point represents a separate study for the indicated association. Horizontal line represents the mean value.

The relevant studies included 38369 individuals (13043 cases and 25326 controls) for rs13361189 variant, 34397 individuals (10924 cases and 23473 controls) for rs4958847 variant, and 16972 individuals (6766 cases and 10206 controls) for rs10065172 variant. We observed a wide spectrum of the rs13361189 C allele and rs4958847 A allele frequencies across different ethnicities. Compared with Europeans controls (8.32%, 95% CI=7.07-9.58), Asian controls carried a higher frequency (35.37%, 95%CI= 31.13-39.60; p=2.5×10^-13^) of rs13361189 C allele. Similar result was observed in rs4958847 (p=1.8×10^-10^). For rs10065172 variant, as only one study carried out in Asians was included, one sample T-test was used to compare the differences of allele frequencies between Asian and European controls (p=9.9×10^-7^). (Figure 2)

**Figure 3:**
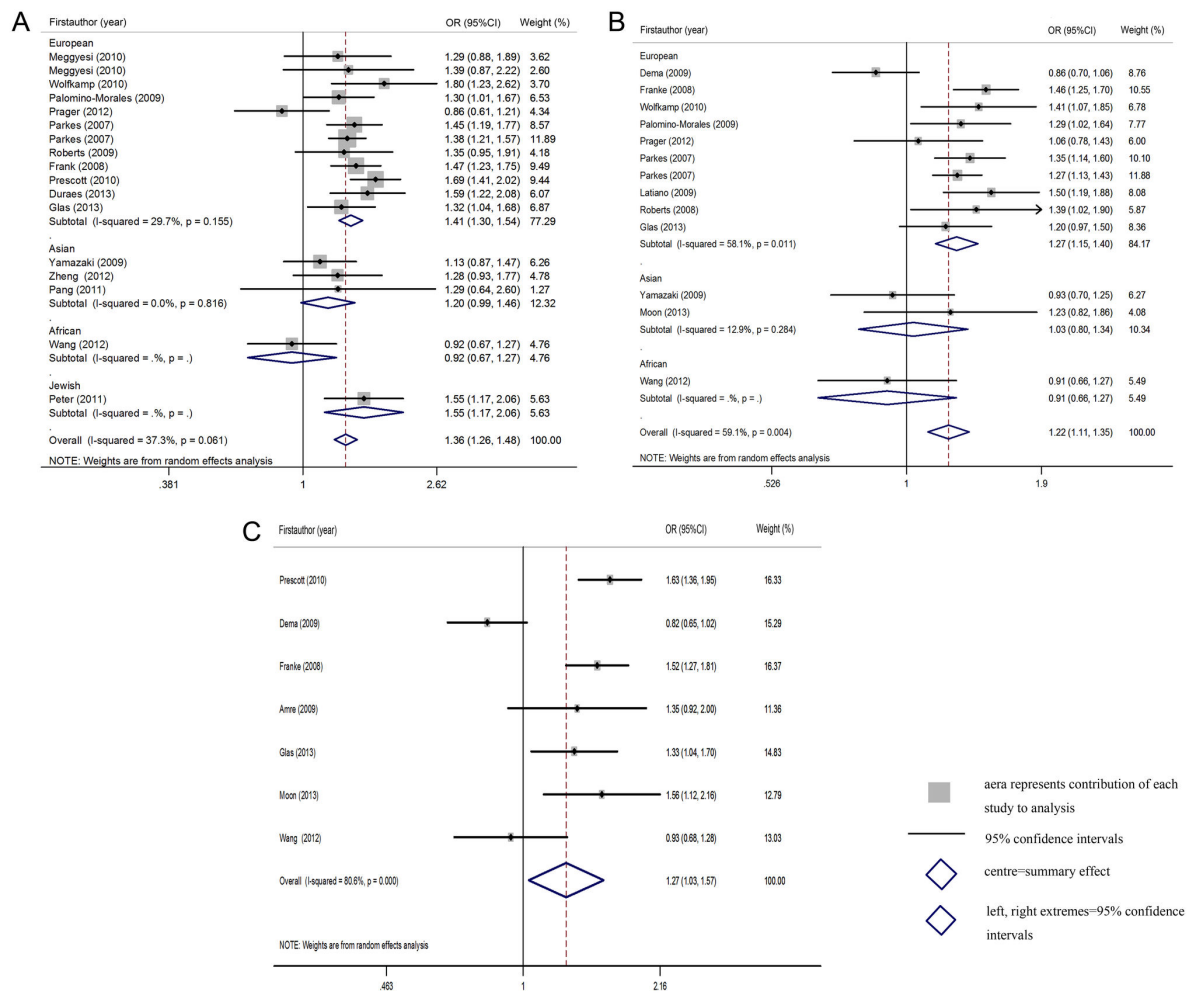
OR estimates with the corresponding 95% CI for the associations between IRGM polymorphisms ((A) rs13361189, (B) rs4958847, and (C) rs10065172) and the risk of CD (dominant model). The sizes of the squares reflect the weighting of included studies; the centre of diamonds reflect summary effect, the left and right extremes of diamonds reflect 95% confidence intervals; CI, confidence interval; OR, odds ratio.

Table 2 showed the meta-analysis results of the association between the allele contrast and genetic models of the different gene polymorphisms and the risk of CD. Significantly elevated CD risk was associated with rs13361189, rs4958847 or rs10065172 for the allele contrast. (OR=1.306 (1.200-1.420), p=5.2×10^-10^, OR=1.182 (1.082-1.290), p=0.0002 and OR=1.248 (1.057-1.473), p=0.009 respectively, significant even after Bonferroni correction). Moreover, significant correlation was also found in the three polymorphisms under other genetic models (homozygote, heterozygote, dominant and recessive models). (Table 2, Figure 3)

**Table 2:** Pooled analysis for the associations between the polymorphism of IRGM and the risk of Crohn’s disease.

Variant	Comparison	Variables	Data NO.	Sameple Size			Test of association		Model	Test of heterogeneity	
				**Case**	**Control**		**OR (95% CI)**	**P-value**		**I^2^ (%) P-value**	
rs13361189	C *vs* T	Overall	18	13043	25326		1.306 (1.200-1.420)	5.2×10^-10^*	R	55.5	0.002
		Europeans	13	11806	24118		1.396 (1.314-1.483)	4.2×10^-27^*	F	35.2	0.101
		Asians	3	868	854		1.084 (0.942-1.248)	0.26	F	0.00	0.984
		All in HWE	15	9483	11364		1.300(1.169-1.446)	1.4×10^-6^*	R	62.5	0.001
	CC *vs* TT	Overall	17	12413	22043		1.565 (1.218-2.010)	0.0004*	R	38.7	0.053
		Europeans	12	11176	20835		2.042 (1.581-2.638)	4.6×10^-8^*	F	11.1	0.336
		Asians	3	868	854		1.064 (0.781-1.450)	0.695	F	0.00	0.763
		All in HWE	15	9483	11364		1.636 (1.220-2.193)	0.001*	R	45.8	0.027
	CT *vs* TT	Overall	17	12413	22043		1.360 (1.279-1.448)	2.8×10^-22^*	F	27.8	0.138
		Europeans	12	11176	20835		1.390 (1.298-1.488)	3.2×10^-21^*	F	21.5	0.232
		Asians	3	868	854		1.239 (1.010-1.520)	0.040	F	0.00	0.550
		All in HWE	15	9483	11364		1.320 (1.198-1.454)	1.9×10^-8^*	R	34.9	0.090
	CC+CT *vs* TT	Overall	17	12413	22043		1.362 (1.255-1.479)	1.3×10^-13^*	R	37.3	0.061
		Europeans	12	11176	20835		1.421 (1.329-1.519)	5.0×10^-25^*	F	29.7	0.155
		Asians	3	868	854		1.199 (0.987-1.455)	0.068	F	0.00	0.816
		All in HWE	15	9483	11364		1.345 (1.214-1.489)	1.3×10^-8^*	R	44.6	0.032
	CC *vs* CT+TT	Overall	17	12413	22043		1.448 (1.129-1.856)	0.004*	R	41.5	0.038
		Europeans	12	11176	20835		1.919 (1.486-2.447)	5.7×10^-7^*	F	9.40	0.353
		Asians	3	868	854		0.941 (0.705-1.256)	0.682	F	0.00	0.478
		All in HWE	15	9483	11364		1.506 (1.128-2.012)	0.006*	R	48.3	0.019
rs4958847	A *vs* G	Overall	14	9854	21892		1.182 (1.082-1.290)	0.0002*	R	66.6	0.0002
		Europeans	11	9016	21068		1.228 (1.117-1.349)	0.00002*	R	64.0	0.002
		Asians	2	737	970		1.062(0.925-1.220)	0.395	F	33.6	0.220
		All in HWE	11	7578	19398		1.195 (1.076-1.328)	0.001*	R	72.1	0.00009
	AA *vs* GG	Overall	13	9224	18609		1.312 (1.045-1.647)	0.019	R	55.7	0.008
		Europeans	10	8386	17785		1.449 (1.084-1.936)	0.012*	R	55.6	0.016
		Asians	2	737	970		1.120 (0.844-1.485)	0.434	F	24.2	0.251
		All in HWE	11	7578	19398		1.373 (1.057-1.783)	0.017	R	61.6	0.004
	AG *vs* GG	Overall	13	9224	18609		1.205 (1.097-1.323)	0.0001*	R	51.2	0.017
		Europeans	10	8386	17785		1.251 (1.138-1.376)	3.8×10^-6^*	R	49.5	0.037
		Asians	2	737	970		0.986 (0.766-1.268)	0.911	F	0.00	0.426
		All in HWE	11	7578	19398		1.192 (1.071-1.326)	0.001*	R	54.6	0.015
	AA+AG *vs* GG	Overall	13	9224	18609		1.220 (1.106-1.347)	0.00007*	R	59.1	0.004
		Europeans	10	8386	17785		1.269 (1.147-1.403)	3.6×10^-6^*	R	58.1	0.011
		Asians	2	737	970		1.028 (0.810-1.304)	0.820	F	12.9	0.284
		All in HWE	11	7578	19398		1.216 (1.085-1.363)	0.001*	R	63.4	0.002
	AA *vs* AG+GG	Overall	13	9224	18609		1.248 (1.028-1.515)	0.025	R	48.7	0.025
		Europeans	10	8386	17785		1.367 (1.037-1.802)	0.026	R	51.7	0.028
		Asians	2	737	970		1.132 (0.912-1.406)	0.260	F	0.00	0.402
		All in HWE	11	7578	19398		1.300 (1.043-1.619)	0.019	R	55.1	0.014
rs10065172	T vs C	Overall	8	5407	8435		1.248 (1.057-1.473)	0.009*	R	79.4	0.00002
		Europeans	6	5053	8081		1.284 (1.055-1.564)	0.013	R	80.3	0.0001
	TT vs CC	Overall	7	4777	5152		1.543 (1.078-2.207)	0.018	R	48.9	0.068
		Europeans	5	4423	4798		1.717 (1.197-2.464)	0.003*	F	12.6	0.334
	TC vs CC	Overall	7	4777	5152		1.244 (1.012-1.530)	0.038	R	78.3	0.0001
		Europeans	5	4423	4798		1.271 (0.988-1.636)	0.062	R	83.2	0.00009
	TT+TC vs CC	Overall	7	4777	5152		1.273 (1.030-1.573)	0.025	R	80.6	0.00003
		Europeans	5	4423	4798		1.298 (1.008-1.673)	0.043	R	84.1	0.00004
	TT vs TC+CC	Overall	7	4777	5152		1.335 (1.078-1.653)	0.008*	F	33.4	0.173
		Europeans	5	4423	4798		1.668 (1.162-2.393)	0.006*	F	0.00	0.463

F: fixed-model; R: random model; *: P value significant even after Bonferroni correction by 3 comparisons (ie 3 compared SNPs, P=0.05/3=0.017); HWE: Hardy-Weinberg equilibrium.

 In the subgroup analysis by ethnicity, for rs13361189 T>C, significantly increased CD risk was found among European populations in the allelic and all genetic models. (CC+CT vs TT: OR=1.421 (1.329-1.519), p=5.0×10^-25^, significant after Bonferroni correction) However, these associations were not observed in the Asian populations (CC+CT vs TT: OR=1.199 (0.987-1.455), p=0.068). Similarly, statistical association was observed between rs4958847 or rs10065172 variant and CD risk among European population. (AA+AG vs GG: OR=1.269 (1.147-1.403), p=3.6×10^-6^, still significant after Bonferroni correction) and TT+TC vs TT: OR=1.298 (1.008-1.673), p=0.043, non-significant after Bonferroni correction) In addition, the OR for rs13361189 T>C was 1.565 (1.218-2.010) in carriers of two risk C alleles compared with non-risk allele carriers (CC vs TT), which was higher than the risk of one T allele carriers (CT vs TT, OR= 1.360 (1.279-1.448)), suggesting a dose–response with increasing number of the variant allele. The same pattern was seen for either rs4958847 or rs10065172 variant.

#### Ulcerative colitis

 Meta-analysis findings of associations between rs13361189 and rs4958847 in the *IRGM* gene and the risk of UC were shown in Table 3. A total of 4847 (5029) UC patients and 6473 (7202) controls for rs13361189 (rs4958847) polymorphism were investigated. No significant association was observed between the polymorphism of rs13361189 and the risk of UC in all comparisons (C vs T: OR=1.088 (0.989-1.198); p=0.083, CC vs TT: OR=1.428 (0.959-2.126), p=0.079; CT vs TT: OR=1.062 (0.955-1.180), p=0.266; dominant model: OR=1.079 (0.973-1.197), p=0.149; and recessive model: OR=1.395 (0.938-2.075), p=0.100.) (Figure S1) Similarly, for rs4955847 variant and UC risk, no obvious association was observed in allelic and genetic models. (Figure S1, Table 3)

**Table 3:** Pooled analysis for the associations between the polymorphisms of IRGM and the risk of ulcerative colitis.

Variant	Comparison	Ethnicity	Data NO.	Sample Size			Test of association		Model	Test of heterogeneity	
				**Case**	**Control**		**OR (95% CI)**	**P-value**		**I^2^ P-value**	
rs13361189	C *vs* T	Overall (Europeans)	8	4564	5512		1.088 (0.989-1.198)	0.083	F	0.00	0.462
	CC *vs* TT	Overall (Europeans)	8	4564	5512		1.428 (0.959-2.126)	0.079	F	5.90	0.385
	CT *vs* TT	Overall (Europeans)	8	4564	5512		1.062 (0.955-1.180)	0.266	F	0.00	0.459
	CC+CT *vs* TT	Overall (Europeans)	8	4564	5512		1.079 (0.973-1.197)	0.149	F	0.00	0.456
	CC *vs* CT+TT	Overall (Europeans)	8	4564	5512		1.395 (0.938-2.075)	0.100	F	7.60	0.372
rs4958847	A *vs* G	Overall	8	4489	5621		1.031 (0.955-1.112)	0.438	F	0.50	0.425
		Europeans	7	4232	5101		1.023 (0.943-1.109)	0.590	F	11.3	0.343
		All in HWE	7	4197	5113		1.028 (0.950-1.112)	0.491	F	13.9	0.324
	AA *vs* GG	Overall	8	4489	5621		1.195 (0.937-1.525)	0.151	F	0.00	0.863
		Europeans	7	4232	5101		1.142 (0.860-1.518)	0.359	F	0.00	0.821
		All in HWE	7	4197	5113		1.250 (0.973-1.607)	0.081	F	0.00	0.975
	AG *vs* GG	Overall	8	4489	5621		1.075 (0.921-1.254)	0.361	R	59.9	0.015
		Europeans	7	4232	5101		1.039 (0.894-1.208)	0.621	R	56.7	0.031
		All in HWE	7	4197	5113		1.057 (0.895-1.248)	0.514	R	62.7	0.013
	AA+AG *vs* GG	Overall	8	4489	5621		1.066 (0.935-1.215)	0.343	R	48.0	0.062
		Europeans	7	4232	5101		1.015 (0.927-1.111)	0.751	F	43.6	0.100
		All in HWE	7	4197	5113		1.058 (0.916-1.222)	0.446	R	53.6	0.044
	AA *vs* AG+GG	Overall	8	4489	5621		1.053 (0.852-1.302)	0.631	F	0.00	0.747
		Europeans	7	4232	5101		1.137 (0.857-1.508)	0.375	F	0.00	0.727
		All in HWE	7	4197	5113		1.086 (0.875-1.348)	0.445	F	0.00	0.848

F: fixed-model; R: random model; HWE: Hardy-Weinberg equilibrium.

### Test of heterogeneity

There was significant heterogeneity in most comparisons of three *IRGM* SNPs in the total analysis of CD. (Table 2) Then meta-regression was carried out to assess the source of heterogeneity for dominant model comparison by year of publication, ethnicity and sample size (individuals more than 500 in both cases and controls). The results showed that ethnicity could explain 41.37% and 19.77% of the τ^2^ in rs13361189 and rs4958847 variants, respectively. Moreover, sample size could explain 63.28% of τ^2^ under dominant model in rs13361189 variant. However, for rs10065172 variant, meta-regression analyses did not show any sources that contribute to the substantial heterogeneity. 

### Sensitivity analyses and cumulative meta-analysis

**Figure 4:**
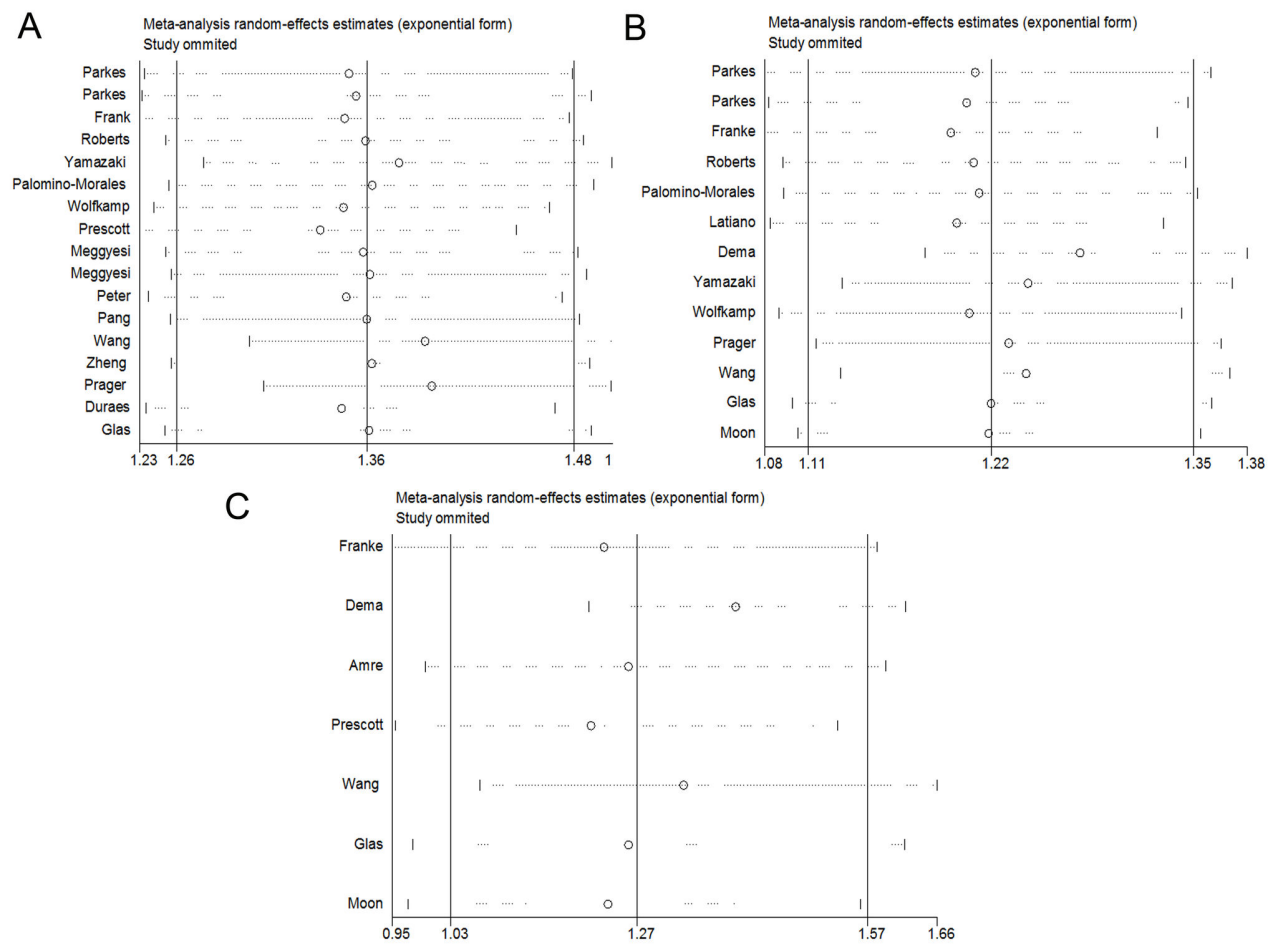
Sensitivity analysis on the associations between IRGM polymorphisms ((A) rs13361189, (B) rs4958847, and (C) rs10065172) and CD risk (dominant model). Results were computed by omitting each study (left column) in turn, Bars: 95% confidence interval.

Sensitivity analyses indicated that the pooled ORs were consistently significant in CD by omitting one study at a time, suggesting robustness of our results. (Figure 4, Figure S2) Although there were two studies (18,21) which deviated from HWE, the corresponding pooled ORs were not materially altered with or without including these two studies in all comparisons. (Table 2, 3)

In addition, sensitivity analyses showed that three independent studies [19,21,32] were the potential origin of heterogeneity in association between rs13361189 variant and CD risk. The heterogeneity was effectively removed by exclusion of these four studies (C vs T: Ph=0.121, CC vs TT: Ph=0.190, CT vs TT: Ph=0.700, CC+CT vs TT: Ph=0.595, and CC vs CT+TT: Ph=0.145). For rs4958847 variant, three studies [20,32,33] were responsible for the heterogeneity. The Q-test of heterogeneity was decreased or removed after exclusion of three studies: A vs G: Ph=0.086, AA vs GG: Ph=0.283, AG vs GG: Ph=0.315, AA+AG vs GG: Ph=0.259, and AA vs AG+GG: Ph=0.409. Two studies [20,33] were the possible sources of heterogeneity of rs10065172, when excluding, the heterogeneity was removed. (T vs C: Ph=0.596, TT vs CC: Ph=0.930, TC vs CC: Ph=0.677, TT+TC vs CC: Ph=0.727, TT vs TC+CC: Ph=0.810)

**Figure 5:**
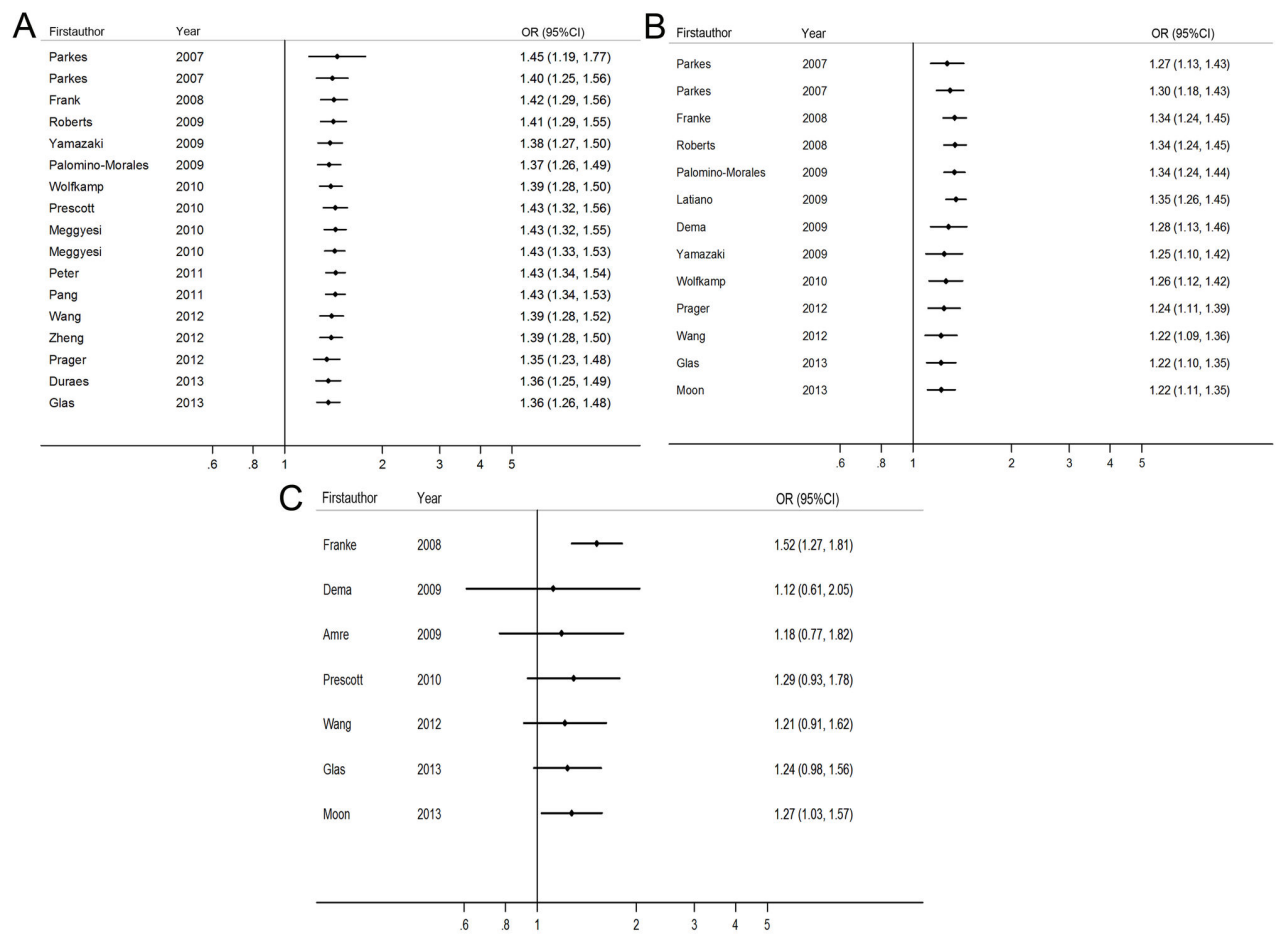
Cumulative meta-analysis: pooled OR with the corresponding 95% CI at the end of each year information step is shown for IRGM polymorphisms ((A) rs13361189, (B) rs4958847, and (C) rs10065172) and risk of CD (dominant model).

In the cumulative meta-analysis, the pooled ORs tended to be stable and the associations tended toward significant with accumulation of more data over time between rs13361189, rs4958847 or rs10065172 polymorphism and CD risk. (Figure 5) However, Figure S3 presented that the associations remained non-significant with accumulation of more data over time in rs13361189 or rs4958847 variant and risk of UC. 

### Publication bias

Funnel plots and Egger’s test were performed to assess publication bias. The shapes of the funnel plots did not reveal evidence of obvious asymmetry in all comparison models. Then, the Egger’s test was used to provide statistical evidence of funnel plot symmetry. Egger’s test did not show any evidence of publication bias of rs13361189 variant (P=0.237 for CC+CT vs TT in CD, P=0.631 for CC+CT vs TT in UC), rs4958847 variant (P=0.278 for AA+AG vs GG in CD, P=0.108 for AA+AG vs GG in UC), or rs10065172 variant (P=0.479 for TT+TC vs CC in CD). Figure S4 and Figure S5 showed the funnel plots of dominant models in the three IRGM SNPs.

## Discussion

IRGM gene, located on chromosome 5q33.1, plays an important role in autophagy. Autophagy has a central function in physiological and pathological processes, involving in innate and adaptive immunity by delivering intracellular pathogens and other antigens. Singh et al demonstrated that IRGM can induce autophagy to eliminate intracellular mycobacterium tuberculosis [41]. Moreover, IRGM 1 -deficient mice have a reduced defense against intracellular pathogens such as *Toxoplasma gondii* and *Listeria monocytogenes* [42]. The presence of bacterial flora is essential for IBD formation in animal models [43]. In addition, autophagy plays an important role in the elimination of apoptotic bodies, and the failure of which might contribute to persistent inflammation in CD [44]. In the recent GWAS meta-analysis, Frank provided strong evidence for the association between IRGM rs7714584 and the risk of IBD (P<10^-8^, meeting genome-wide significance). Parkes et al [18] found two immediately flanking *IRGM* variants (rs13361189 and rs4958847) associated with CD in two different British cohorts. Thus, *IRGM* may appear to be a good candidate for IBD. However, the results of associations between three *IRGM* variants (rs13361189, rs4958847 and rs10065172) and risk of IBD were contradictory. Therefore, we saw the need to perform pooled analyses with larger sample size by summarizing previous case-control studies in order to understand the association between IRGM variants and IBD risk better.

Our meta-analysis showed significant susceptibility of CD from rs13361189 in the overall population in all genetic contrasts. When stratified by ethnicity, a significant association with rs13361189 was observed in European population, but not in Asians. Similar results were also found between the rs4958847 or rs10065172 variant and risk of CD in overall and European population. It is widely accepted that genetic markers in predisposition to IBD vary across ethnic groups. For instance, nucleotide oligomerization domain 2 (NOD2) polymorphisms have been strongly association with CD in Europeans [45,46], but not in Asian population [47-49]. These results suggested that rs13361189 and rs4958847 variants might be ethnic population-specific risk factors for CD. However, the lack of association in the Asian population from this study might not be very conclusive owing to the relatively small number of Asian populations used in the analysis (only 2 studies for rs4955847 and 3 studies for rs13361189 in Asian population). Therefore, further studies in Asian populations with larger sample sizes might need to be performed to clarify possible roles of IRGM polymorphisms in CD. Since these SNPs are close to each other, we used 1000 Genomes Pilot sequence data to identify whether these SNPs were in linkage disequilibrium (LD) (r^2^>0.8). The results showed that rs13361189 was in perfect LD with 2 other IRGM SNPs (rs10065172 and rs1000113, r^2^=1.000) in Europeans. However, rs4958847 was not in LD with 3 other IRGM SNPs (rs13361189, rs10065172 and rs1000113, r^2^=0.304). 

Overall, no significant association between rs13361189 or rs4958847variant and susceptibility to UC was found in this meta-analysis in any genetic model. To date, there was lack of association of these two SNPs with UC in all the individual studies. Recently, twin studies and familial clustering of cases suggested that genetic factors were likely to play a more prominent role in CD than in UC [3]. This observation was also supported by the finding that both *NOD2* and *ATG16L1* were associated with CD, but not with UC [50,51]. 

Heterogeneity was significant for the most comparisons of rs13361189 polymorphism in overall population. To identify the source of heterogeneity, meta-regression and subgroup analysis were carried out. We found that ethnicity was identified as a potential source of between-study heterogeneity. Meta-regression indicated that ethnicity could explain 41.37% of τ^2^. Moreover, the heterogeneity was remarkably decreased among Asian and European population, (CC vs TT: P=0.763, P=0.336, respectively), which may be attributed that IBD is a complex disease and different genetic backgrounds or different environments existed among different ethnicities. Moreover, the sample size could explain 63.28% of τ^2^ under dominant model. In addition, the pooled OR did not change in the sensitivity analysis by excluding studies departed from HWE.

Our meta-analysis significantly increased statistical power by pooling data from different studies, while several limitations should be considered in the present meta-analysis. First, only 2 and 3 studies were performed in Asians for rs4955847 and rs13361189 variants, respectively. Therefore validation of association is required in other population. Second, significant heterogeneity between studies was detected in the current meta-analysis, whereas difference in ethnicity was identified as potential sources of heterogeneity. Third, gene–environment and gene-gene interactions were not analyzed because of insufficient data. 

In conclusion, despite these limitations, our meta-analysis still yields statistically significant results. The present data synthesis indicated that rs13361189, rs4958847 and rs10065172 were considered to be risk factors of CD in Europeans but not of UC. In addition, subgroups analysis suggested that this increased risk may be ethno-specific. Further studies in other ethnic groups (e.g. Asians and Africans) are needed to clarify possible roles of *IRGM* polymorphisms in CD or UC. To identify the exact role of *IRGM* polymorphisms in the pathogenesis of CD, more studies such as animal disease modeling are of great importance.

## Supporting Information

Checklist S1 **PRISMA checklist.**
(DOC)

Figure S1 **OR estimates with the corresponding 95% CI for the associations between IRGM polymorphisms ((A) rs13361189 and (B) rs4958847) and the risk of UC (dominate model).**
(TIF)

Figure S2 **Sensitivity analysis on the associations between IRGM polymorphisms ((A) rs13361189 and (B) rs4958847) and UC risk (dominate model).**
(TIF)

Figure S3 **Cumulative meta-analysis: pooled OR with the corresponding 95% CI at the end of each year information step is shown for IRGM polymorphisms ((A) rs13361189 and (B) rs4958847) and risk of UC (dominate model).**
(TIF)

Figure S4 **Funnel plots of the association between IRGM polymorphisms ((A) rs13361189, (B) rs4958847, and (C) rs10065172) and CD risk (dominant model).**
(TIF)

Figure S5 **Funnel plots of the association between IRGM polymorphisms ((A) rs13361189 and (B) rs4958847) and UC risk (dominate model).**
(TIF)
