# Supplementary figures and images for: Association between Variants of the Autophagy Related Gene – IRGM and Susceptibility to Crohn’s Disease and Ulcerative Colitis: A Meta-Analysis
Source: PLoS One. 2013 Nov 13;8(11):e80602. doi: 10.1371/journal.pone.0080602 (PMC3827440; doi:10.1371/journal.pone.0080602)

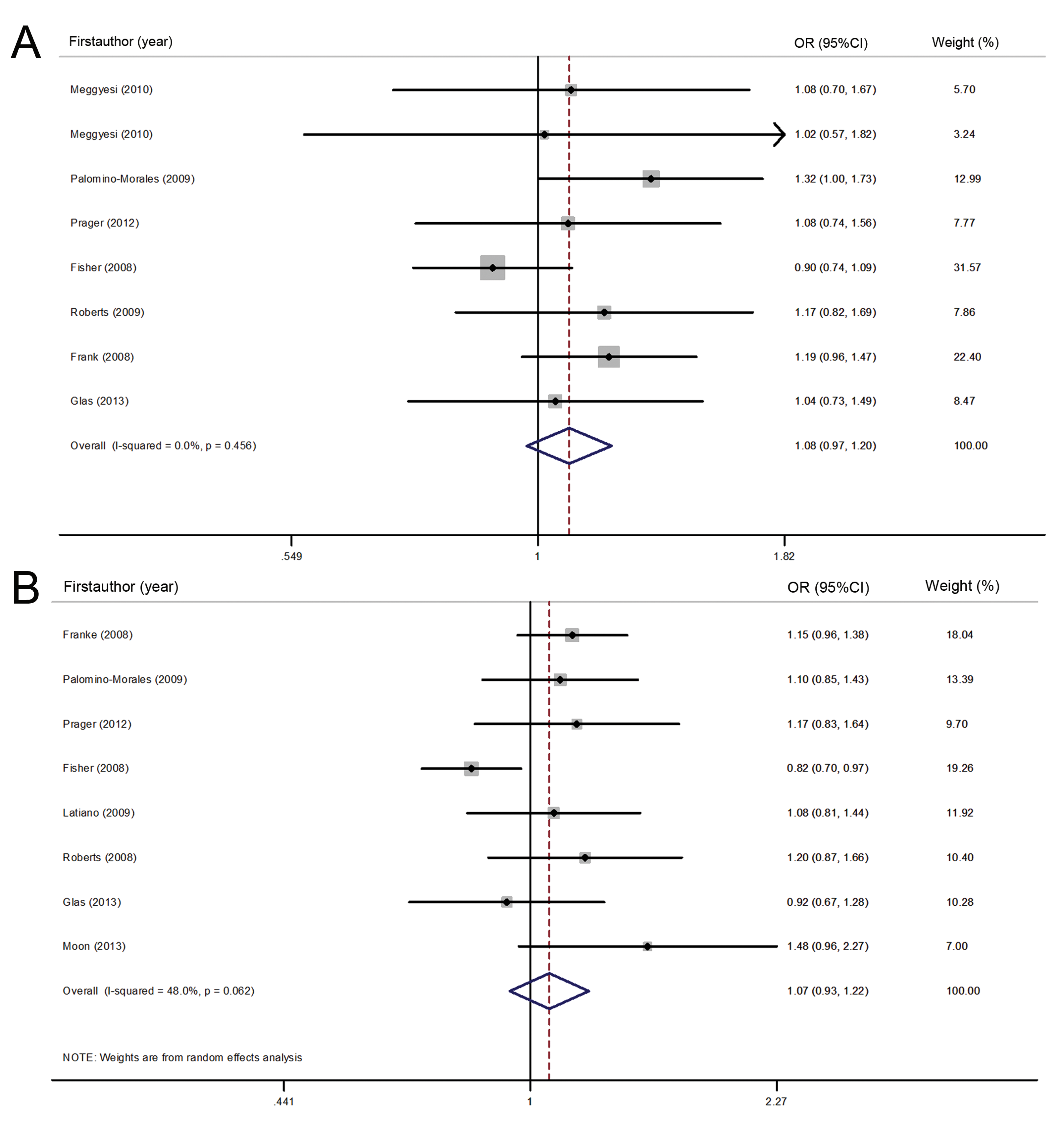

Supplement: Figure S1 — OR estimates with the corresponding 95% CI for the associations between IRGM polymorphisms ((A) rs13361189 and (B) rs4958847) and the risk of UC (dominate model). (TIF) [file pone.0080602.s002.tif]

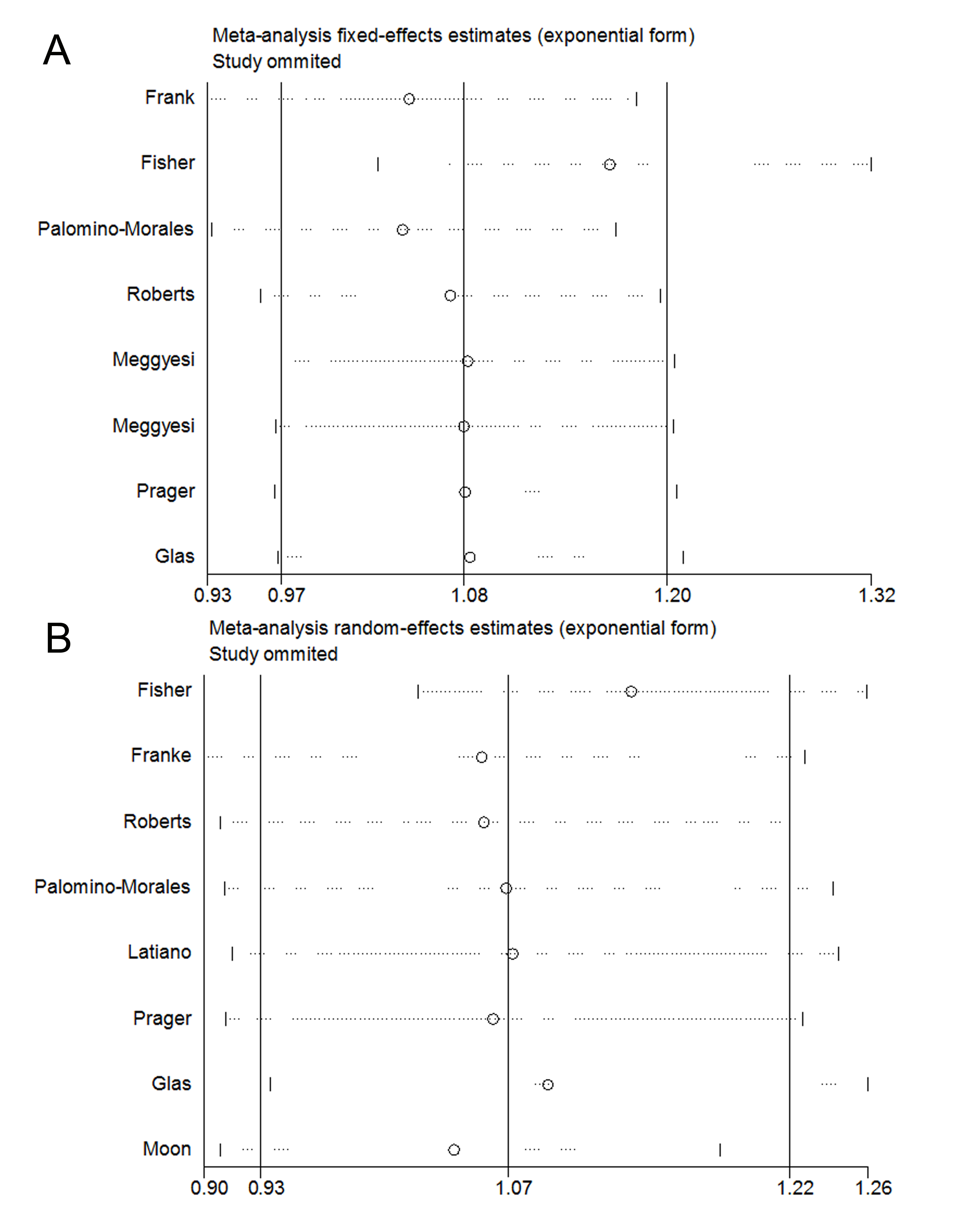

Supplement: Figure S2 — Sensitivity analysis on the associations between IRGM polymorphisms ((A) rs13361189 and (B) rs4958847) and UC risk (dominate model). (TIF) [file pone.0080602.s003.tif]

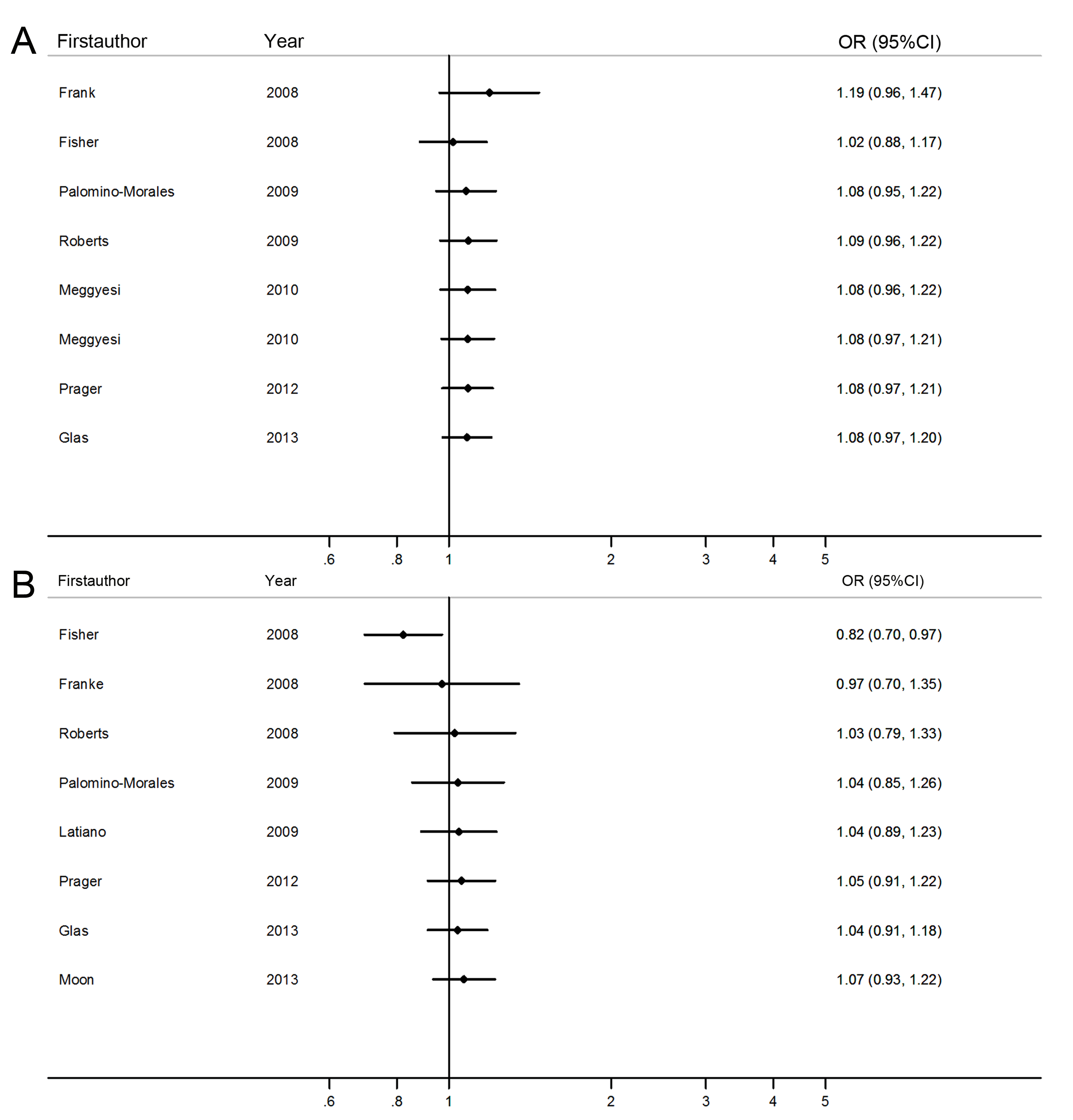

Supplement: Figure S3 — Cumulative meta-analysis: pooled OR with the corresponding 95% CI at the end of each year information step is shown for IRGM polymorphisms ((A) rs13361189 and (B) rs4958847) and risk of UC (dominate model). (TIF) [file pone.0080602.s004.tif]

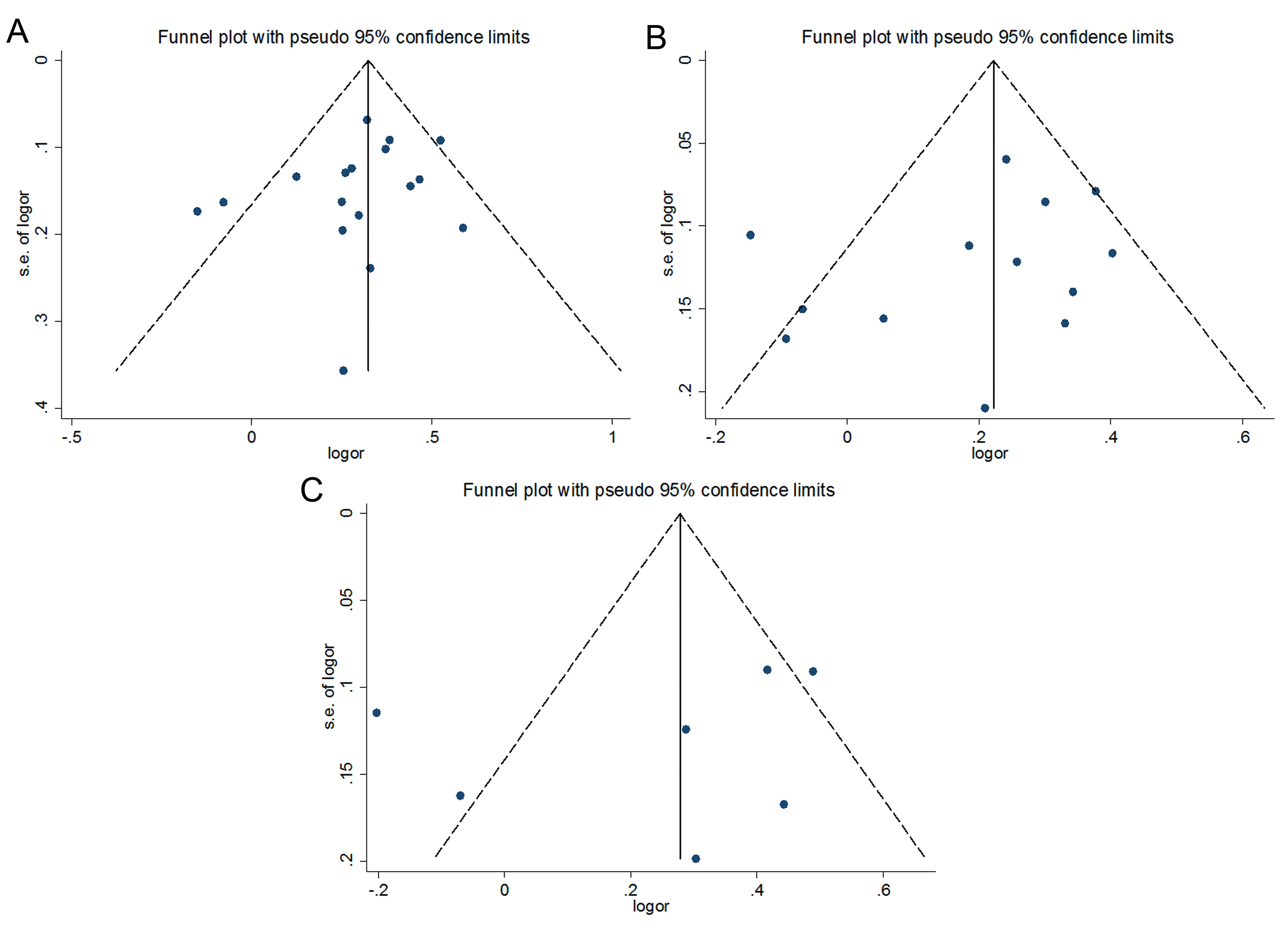

Supplement: Figure S4 — Funnel plots of the association between IRGM polymorphisms ((A) rs13361189, (B) rs4958847, and (C) rs10065172) and CD risk (dominant model). (TIF) [file pone.0080602.s005.tif]

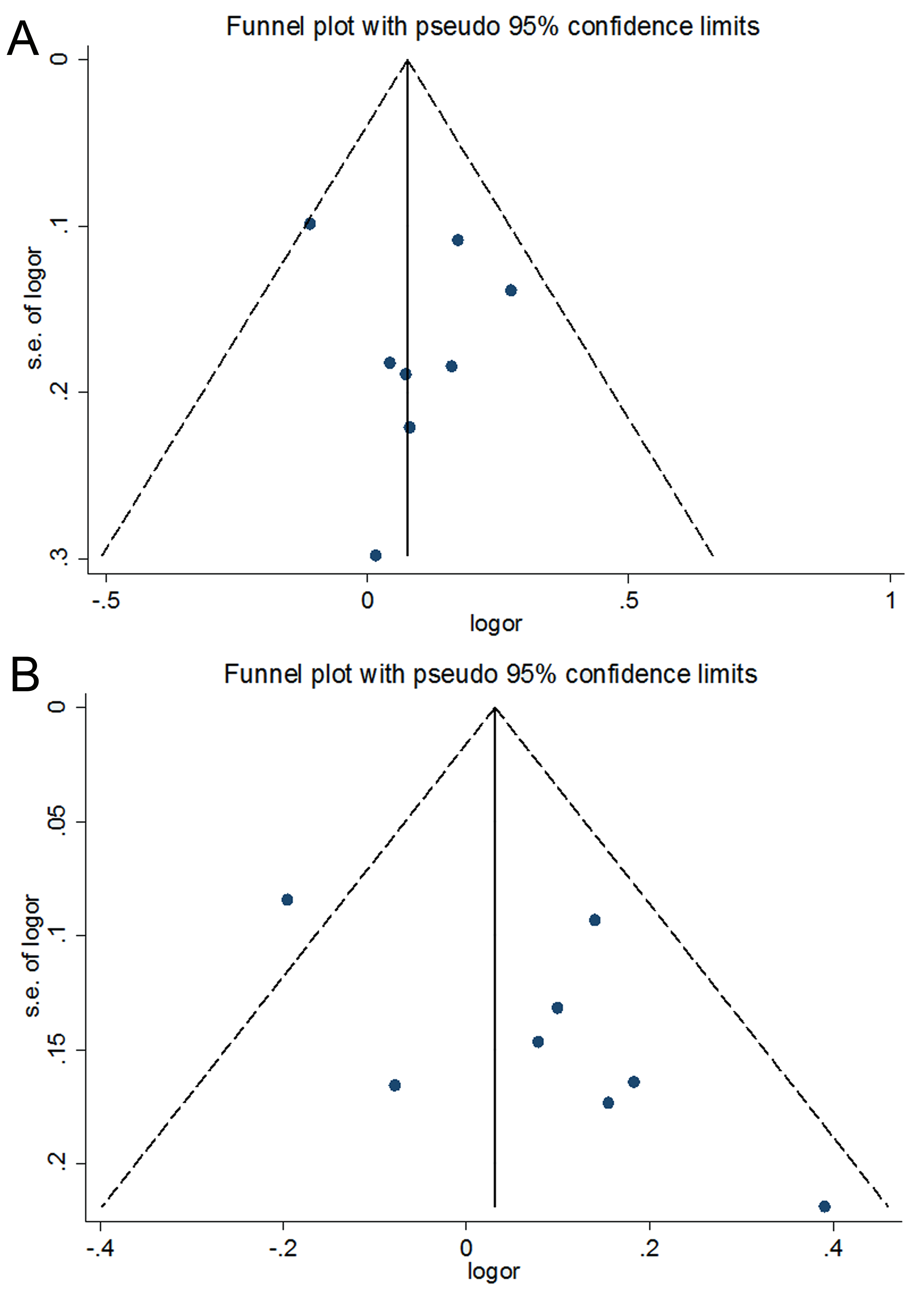

Supplement: Figure S5 — Funnel plots of the association between IRGM polymorphisms ((A) rs13361189 and (B) rs4958847) and UC risk (dominate model). (TIF) [file pone.0080602.s006.tif]
